# The analysis of biodistribution and tumor uptake of [^18^F]AlF-FAPI-74 in patients with soft tissue sarcoma and gastro-intestinal tumors compared with [^18^F]FDG in a prospective, exploratory study

**DOI:** 10.1186/s41824-026-00295-7

**Published:** 2026-03-06

**Authors:** Emil Novruzov, Frederik L. Giesel, Eduards Mamlins, Tadashi Watabe, Jens Cardinale, Christina Antke, Yuriko Mori, Nils Schupps, Claudio Pinto, Cristian Soza-Ried, Rene Fernandez, Horacio Amaral, Vasko Kramer, Leonardo Badinez

**Affiliations:** 1 Department of Nuclear Medicine, Medical Faculty, University Hospital Duesseldorf, Heinrich-Heine-University Duesseldorf, Moorenstrasse 5, 40225 Düsseldorf, Germany; 2 Department of Nuclear Medicine and Tracer Kinetics, Graduate School of Medicine, Osaka University, Osaka, Japan; 3 Institute for Radiation Sciences, Osaka University, Osaka, Japan; 4 Departamento Anatomia Patologica, Hospital Sotero del Rio, Santiago, 8207257 Chile; 5 Center for Nuclear Medicine, PET/CT Positronmed, Santiago, 7501068 Chile; 6 Positronpharma SA, Santiago, 7501068 Chile; 7 Facultad de Medicina Veterinaria y Agronomía, Instituto de Ciencias Naturales, Universidad de las Américas, Santiago, Chile; 8 Instituto Radiooncológico Santiago INRAD, Santiago, 7750000 Chile

**Keywords:** [^18^F]AlF-FAPI-74, FAPI-74 PET, ^18^F-labelling, Gastrointestinal tumors, Colorectal carcinoma, Soft tissue tumors, Pancreatic carcinoma

## Abstract

**Background:**

The roll-out of FAP (fibroblast-activation-protein) imaging with small-molecule PET tracers marks one of the major breakthroughs in nuclear medicine. The majority of current evidence, however, has been generated by ^68^Ga-labelled FAPI tracers, a reliance that presents serious economical and logistic challenges to the oncological community. Hence, current research focuses on development of ^18^F-labelled FAPI tracers to overcome the limitations of ^68^Ga-labelling. [^18^F]AlF-FAPI-74, a recently introduced radiotracer, emerges as a promising candidate to fulfill those unmet clinical needs. This single-center, exploratory study sought to investigate the biodistribution and tumor uptake of [^18^F]AlF-FAPI-74 across various tumor entities in a head-to-head comparison with [^18^F]FDG imaging.

**Material and method:**

A total of 19 patients (15 males and 4 females) were enrolled in this prospective study with a head-to-head analysis of [^18^F]FAPI-74 and [^18^F]FDG PET/CT imaging between May 2021 and January 2024. According to inclusion criteria, patients underwent PET/CT scans, when they had either a biopsy-proven or highly suspected malignancy with a prior FDG imaging, or highly suspected or proven recurrence by clinical or other imaging findings or therapy monitoring after neoadjuvant or also palliative radiochemotherapy. All patients had histologically proven malignancies of pancreatic adenocarcinoma (PDAC) in 8 patients, soft tissue sarcoma (STS) in 6 patients, colorectal carcinoma (CRC) in 4 patients and gastric carcinoma in 1 patient. The mean injected activity for [^18^F]FAPI-74 and [^18^F]FDG PET/CT was 239 MBq (± 57) and 209 MBq (± 74) and the mean uptake time was 72 min (± 10) and 73 min (± 17), respectively.

**Results:**

[^18^F]AlF-FAPI-74 demonstrated superior imaging characteristics compared to [^18^F]FDG, identifying a greater number of both primary and metastatic lesions across all tumor entities. Our SUV-metrics analysis revealed more favorable results for the detection of primary lesions with [^18^F]FAPI-74 imaging (e.g. SUV_max_ ± SD, 9.79 ± 4.2 vs. 6.41 ± 2.8, [^18^F]FAPI-74 vs. [^18^F]FDG, respectively), whereas only the TBR in relation to blood pool appeared to be statistically significant (5.08 vs. 3.15 (p: 0.03); [^18^F]FAPI-74 vs. [^18^F]FDG, respectively). We observed a similar tendency in the analysis of SUV-metrics (e.g. SUV_max_ ± SD, 7.42 ± 2.8 vs. 6.18 ± 2.7, [^18^F]FAPI-74 vs. [^18^F]FDG, respectively) in metastatic lesions as well.

**Conclusions:**

In this prospective, exploratory study cohort, [^18^F]FAPI-74 appeared to display more favorable imaging characteristics such as lesion contrast and delineation compared with [^18^F]FDG. Given the advantages of ^18^F-labeling, [^18^F]FAPI-74 warrants further investigation with larger cohorts to determine its diagnostic potential and clinical impact.

**Supplementary Information:**

The online version contains supplementary material available at 10.1186/s41824-026-00295-7.

## Introduction

FAP (fibroblast-activation-protein) imaging with small-molecule PET tracers marks one of the major breakthroughs in nuclear medicine. Over 90% of epithelial malignancies upregulate FAP expression with increasing tumor aggressivity and invasiveness. FAP is a type II membrane-bound glycoprotein with dipeptidyl peptidase and endopeptidase activity, highly expressed on cancer-associated-fibroblasts (CAFs), for dynamic remodeling effects of tumor microenvironment in epithelial cancers, most soft tissue sarcomas as well as in certain benign tissue/processes such as granulation tissue of wound healing and certain fetal mesenchymal fibroblasts. However, resting fibroblasts or normal tissue do not express FAP. FAP expression in tumor stroma plays a major role in modulation of tumor growth and invasiveness by a complex interaction with tumor cells (Fitzgerald and Weiner 2020, Kratochwil et al. 2019).

In addition, few studies implicate FAP not to be only a stromal target, but also expressed on cell membranes of certain epithelial tumor cells such as pancreatic ductal adenocarcinoma, gastric cancer, soft tissue sarcomas and colorectal cancer (Shi et al. 2012, Mori et al. 2004, Iwasa et al. 2003, Crane et al. 2023). In fact, previous studies demonstrated an overall comparable or even superior diagnostic performance of FAPI (fibroblast-activation-protein-inhibitor) PET for the abovementioned tumor entities compared with [^18^F]FDG. Subsequently, this unique FAP expression pattern on these tumor entities may further enhance the uses of FAP imaging for therapy monitoring and FAP-targeted radioligand therapy (Hu et al. 2025, Gao et al. 2024, Zhuang et al. 2024, Kessler 2023, Giammarile et al. 2024, Yun et al. 2024).

Since its introduction, the medical translation, either in research setting or regular clinical care, has been predominantly conducted by ^68^Ga-labelled tracers, even though there are also some early results with ^99m^Tc-labelled FAP-SPECT tracers. ^68^Ga-labelling has a number of traditional shortcomings including limited batch production of approximately 2–3 patient doses per generator-elution with a subsequent reduced cost-effectiveness, and also a restricted spatial resolution. Additionally, the short half-life (68 min, 1.90-MeV positron energy) of ^68^Ga necessitates an in-house production that prevents the establishment of long-distance supply-chains for the nuclear medicine facilities without appropriate infrastructure. This represents a remarkable obstacle for a widespread, economically profitable deployment of these PET-Tracers (Loktev et al. 2018, Lindner et al. 2018, Lindner et al. 2020, Giesel et al. 2021, Glatting et al. 2022).

**Fig. 1:**
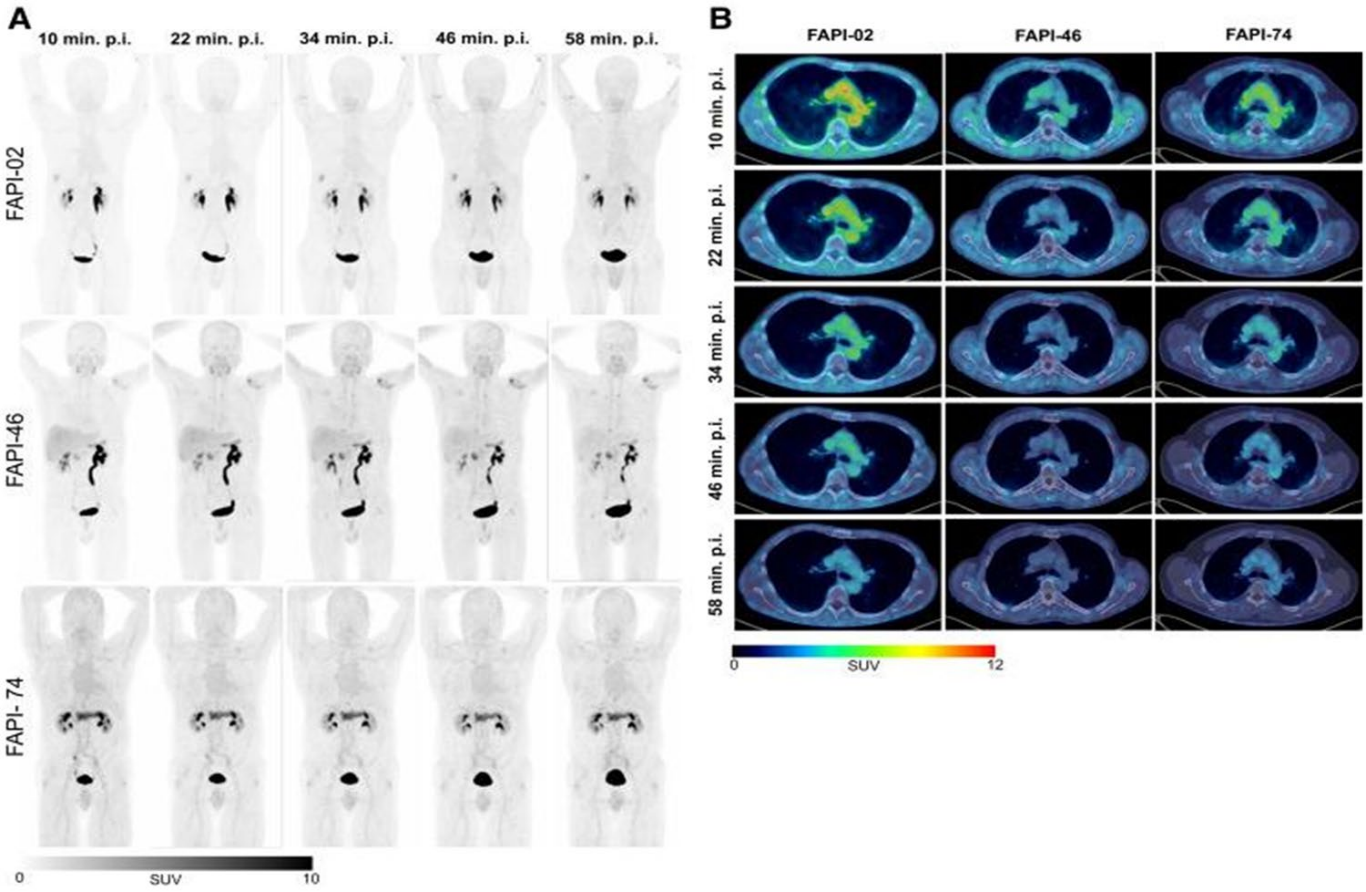
Representative maximum-injection projections of various radiolabeled FAP-ligands including FAPI-74, adapted from (Glatting et al. 2022)

Thus, one of the focuses of the current FAPI research concentrates on the development and analysis of ^18^F-labelled FAPI-PET tracers. ^18^F-labelling could overcome these challenges owing to its favorable physical properties such as a longer half-life (109 min) and lower positron energy (0.65-MeV), leading to a better spatial resolution and much better cost-effectiveness due to large-scale tracer production and elimination of the need for an on-site radiochemistry for nuclear medicine facilities. To this end, the Heidelberg research group introduced the [^18^F]AlF-FAPI-74 ([^18^F]FAPI-74) as a highly-promising counterpart to the existing ^68^Ga-labelled FAP-PET tracers (Giesel et al. 2021). The authors underscored its superior diagnostic features and its more favorable profile of radiation exposure for patients, albeit its ground structure does not allow a theranostic deployment (Giesel et al. 2021, Lindner et al. 2021). A comparative overview of tracer biodistribution of FAPI ligands is illustrated in Fig. 1 (Glatting et al. 2022). However, there is only very scarce data in the literature about the biodistribution and tumor uptake of [^18^F]FAPI-74 across various malignancies.

We sought to report the final results of our single-center, prospective, exploratory study involving the head-to-head comparison of [^18^F]FAPI-74 and [^18^F]FDG PET/CT scans across various tumor entities.

## Method and materials

### Clinical study design and patient cohort

A total of 19 patients with a male patient predominance (15 males and 4 females) were enrolled in this prospective study with a head-to-head analysis of [^18^F]FAPI-74 and [^18^F]FDG PET/CT imaging between May 2021 and January 2024 (Table 1). The interim-results of this study were already published which involved the ad-hoc analysis of seven patients with pancreatic cancer (Novruzov et al. 2023). The inclusion criteria were as follows:

- Biopsy-proven or highly suspected gastro-intestinal malignancy and/or soft tissue sarcoma with a prior FDG imaging.
- Highly suspected or proven recurrence by clinical or other imaging findings.
- Therapy monitoring after neoadjuvant or also palliative radiochemotherapy.

The pediatric patients or female patients with suspected pregnancy were excluded. PET scans were performed according to clinical standard-of-care indications (approved by the regional ethics committee board (CEC SSM Oriente/19062020)). The study was conducted in accordance with the Declaration of Helsinki, Good Clinical Practices, and national regulations. Oral and written informed consent were obtained from all patients on an individual-patient basis following national regulations.

### Radiochemistry and image acquisition

The precursor material was obtained from SOFIE and [^18^F]FAPI-74 solution was synthesized in accordance with local GMP regulations using a procedure modified from Giesel et al. (Giesel et al. 2021). The patients required no special preparation for the conduct of [^18^F]FAPI-74 PET/CT scan, whereas each patient had to follow instructions for [^18^F]FDG PET/CT to fast at least 4 h and also have blood glucose levels of < 150 mg/dl. All participants underwent both [^18^F]FAPI-74 and [^18^F]FDG PET/CT whole-body scans using the same PET/CT scanner (Biograph Vision or Biograph Flow mCT20; Siemens, Erlangen, Germany) with interchangeable acquisition protocols within a time interval of 11 (± 5) days (range 1–20 days) and without any substantial change in treatment during the interval (Supplementary Table). The [^18^F]FDG scans were performed using contrast-enhanced CT followed by [^18^F]FAPI-74 PET/CT scans using low-dose CT for anatomical localization and attenuation correction.

The mean injected activity for [^18^F]FAPI-74 and [^18^F]FDG PET/CT was 239 MBq (± 57) and 209 MBq (± 74) and the mean uptake time was 72 min (± 10) and 73 min (± 17), respectively. Vital signs were monitored for all patients before and at the end of the intervention with no observed adverse events or drug reactions.

### Image analysis

Circular regions of interest (ROI) were placed over the normal organs by investigator from University Hospital Düsseldorf (EN; supervised by EM & FLG). For small organs (thyroid, parotid gland, myocardium, oral mucosa, and spinal cord) a diameter of 1 cm was used. For larger organs (brain, muscle, liver, spleen, kidney, fat, aortic lumen, and lung) a larger (2 cm) ROI was used. Each ROI was automatically incorporated into a 3-dimensional volume of interest with a 40% iso-contouring approach Image analysis was performed via a dedicated software package (Hermes, Affinity 3.0.5; Hermes Medical Solutions, Stockholm, Sweden). All lesions with a significant tracer uptake on the [^18^F]FAPI-74 and [^18^F]FDG scans or unequivocally morphological findings on enhanced CT, whether primary or metastatic, were considered suitable for further analysis. In the case of patients with disseminated metastases, only up to five index lesions were considered for further analysis. The lesions with a significant tracer uptake were classified as such when the SUV_max_ were more than two times that of TBR or they were detectable on MIP (maximum intensity projection). Tumor-to-background ratio (TBR) was derived by dividing the SUV_max_ of tumor lesions by the SUV_max_ of adipose tissue and skeletal muscle in the gluteal region and blood pool in the descending aorta. All the primary lesions were biopsy-proven and the suspected metastatic lesions were confirmed by composite reference method, which included unequivocal correlate on further follow-up imaging or change in clinical course in terms of therapy response.

### Statistical analysis

We used descriptive analyses for demographics, tumor characteristics, and tracer uptake. The statistical analyses were performed via Excel Version 2311 (Microsoft^®^ Excel^®^ 2021 MSO) and SigmaPlot 11.0 (Systat Software Inc., San Jose, CA, USA) and graphical visualization by using SigmaStat Version 3.5 (Systat Software, Inc., San Jose, CA, USA). Comparison between [^18^F]FAPI-74 and [^18^F]FDG PET/CT-SUV metrics in tumor and normal tissue as well as TBRs were performed by paired t-test and Wilcoxon–Mann–Whitney Test. A p value of < 0.05 was considered statistically significant. Data cleaning was performed to identify and correct any errors, inconsistencies, or missing values in the dataset, thus improving the overall quality and reliability of the data.

## Results

### Patient cohort

The study cohort included 4 females and 15 males, mean age of 61 (± 10), with heterogeneous tumor entities, depicted in Table 1. All patients had histologically proven malignancies of pancreatic adenocarcinoma (PDAC) in 8 patients, soft tissue sarcoma (STS) in 6 patients, colorectal carcinoma (CRC) in 4 patients and gastric carcinoma in 1 patient. All patients underwent dual-tracer imaging to complete the diagnostic work-up of primary staging due to an advanced stage of disease at initial diagnosis or highly clinical suspicion of recurrence or therapy response monitoring following a chemoradiotherapy in the setting of (neo-)adjuvant therapy. All patients tolerated the examinations well with no sign of [^18^F]FAPI-74-related adverse effect or complication.

Given the insidious-onset and rapid local invasion, the patients with PDAC underwent more often dual-tracer imaging in the primary staging setting. In total, 4 patients underwent dual-tracer imaging due to suspected recurrence, while 10 patients required PET scans for the therapy response control or re-staging-work-up following chemoradiotherapy.

### Biodistribution of [^18^F]FAPI-74 in normal tissue

**Fig. 2:**
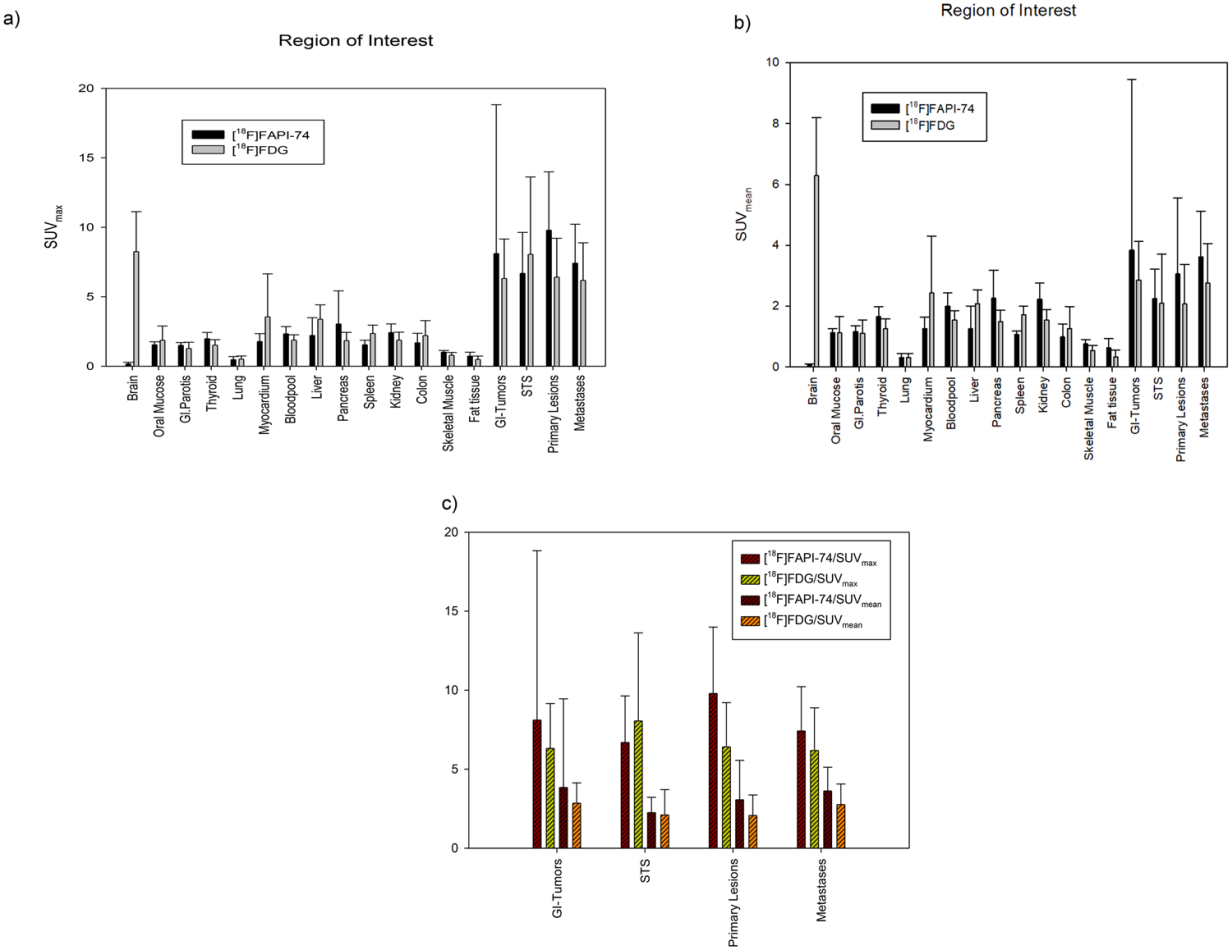
Analysis of biodistribution and tumor uptake of the patient cohort with different malignancies based on intra-individual comparison of [^18^F]FDG and [^18^F]FAPI-74 in terms of SUV_max_ and SUV_mean_ (**a**, **b**). An overview of tumor uptake across different tumor entities (***c***). *STS: soft tissue sarcoma. *GI-Tumor: Gastro-intestinal tumor

The biodistribution of [^18^F]FAPI-74 resembles the pattern with ^68^Ga-labelled FAP ligands, as its activity remains low in normal/background tissue. Moreover, the likely sites of metastatic spread are mostly physiological active tissue with a relevant uptake of [^18^F]FDG. Therefore, [^18^F]FAPI-74 imaging offers a major advantage over [^18^F]FDG for better detectability and delineation of malignant lesions especially in sites with a relatively high glucose activity. Figure 2 illustrates the mean [^18^F]FDG and [^18^F]FAPI-74 uptake in normal organs and malignant lesions 60 min after intravenous tracer administration in a comparative manner.

**Table 1:** Patient characteristics and an overview of clinical indications for the conduct of [^18^F]FAPI-74 and [^18^F]FDG PET/CT scans

Malignancy	Age	Gender	Previous Chemotherapy	Previous Radiotherapy	Previous Surgery	Clinical Indication	Staging Change
PDAC-1	57	F	No	No	No	Primary Staging	No
PDAC-2	69	M	Yes	No	No	Therapy Response/Re-Staging	Yes
PDAC-3	65	M	Yes	Yes	No	Therapy Response/Re-Staging	No
PDAC-4	66	M	Yes	Yes	No	Therapy Response/Re-Staging	No
PDAC-5	78	M	Yes	Yes	No	Therapy Response/Re-Staging	No
PDAC-6	79	M	No	No	No	Primary Staging	No
PDAC-7	75	M	No	No	No	Primary Staging	No
PDAC-8	56	M	Yes	Yes	Yes	Therapy Response/Re-Staging	Yes
STS-1	37	M	No	No	No	Primary Staging	No
STS-2	64	F	Yes	Yes	Yes	Re-Staging	No
STS-3	33	M	Yes	Yes	Yes	Re-Staging	No
STS-4	61	F	Yes	Yes	Yes	Therapy Response/Re-Staging	No
STS-5	52	F	No	Yes	Yes	Therapy Response/Re-Staging	No
STS-6	61	M	Yes	Yes	Yes	Therapy Response/Re-Staging	Yes
GC-1	41	M	Yes	No	Yes	Therapy Response/Re-Staging	No
CRC-1	59	M	Yes	Yes	No	Therapy Response/Re-Staging	No
CRC-2	53	M	yes	no	Yes	Re-Staging	No
CRC-3	73	M	Yes	Yes	Yes	Re-Staging	No
CRC-4	60	F	No	No	No	Primary Staging	Yes

### [^18^F]FAPI-74 uptake in tumor lesions

**Fig. 3:**
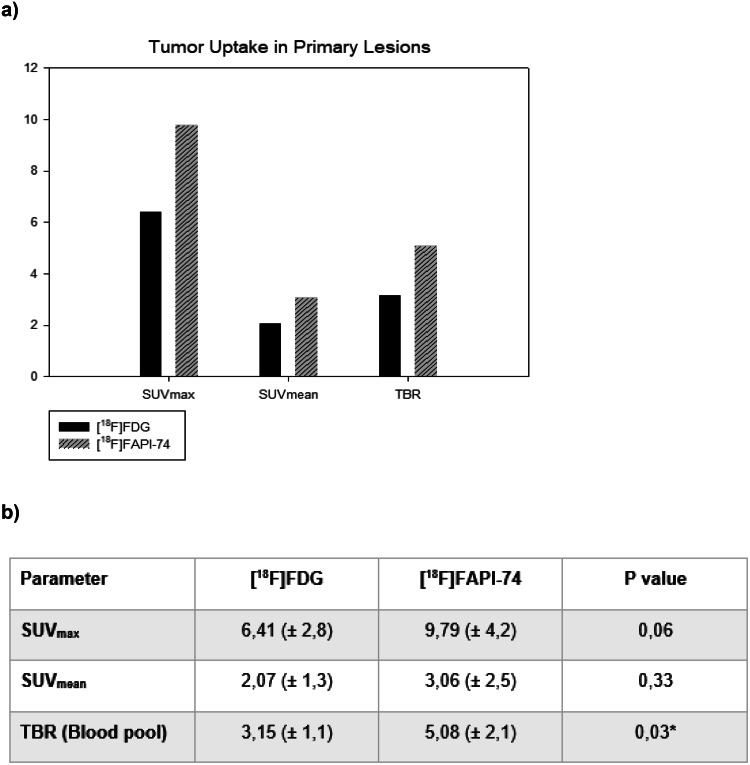
Tumor uptake of [^18^F]FAPI-74 and [^18^F]FDG for the primary lesions after the comparative assessment of mean values of SUV-metrics (**a**). Even though all parameters indicated a more favorable outcome for [^18^F]FAPI-74, TBR was the only statistically significant parameter (**b**). * statistically significant

[^18^F]FAPI-74 revealed more primary and metastatic lesions for tumor entities in total, but also for GI-tumors separately, than [^18^F]FDG. The detection rate of primary lesions in patients with STS was comparable for both tracers, albeit [^18^F]FAPI-74 imaging outperformed [^18^F]FDG regarding metastatic lesions. Our SUV-metrics analysis revealed more favorable results for the detection of primary lesions with [^18^F]FAPI-74 imaging (e.g. SUV_max_ ± SD, 9.79 ± 4.2 vs. 6.41 ± 2.8, [^18^F]FAPI-74 vs. [^18^F]FDG, respectively), whereas only the TBR in relation to blood pool appeared to be statistically significant (5.08 vs. 3.15 (p: 0.03); [^18^F]FAPI-74 vs. [^18^F]FDG, respectively) (Fig. 3a & b).

**Fig. 4:**
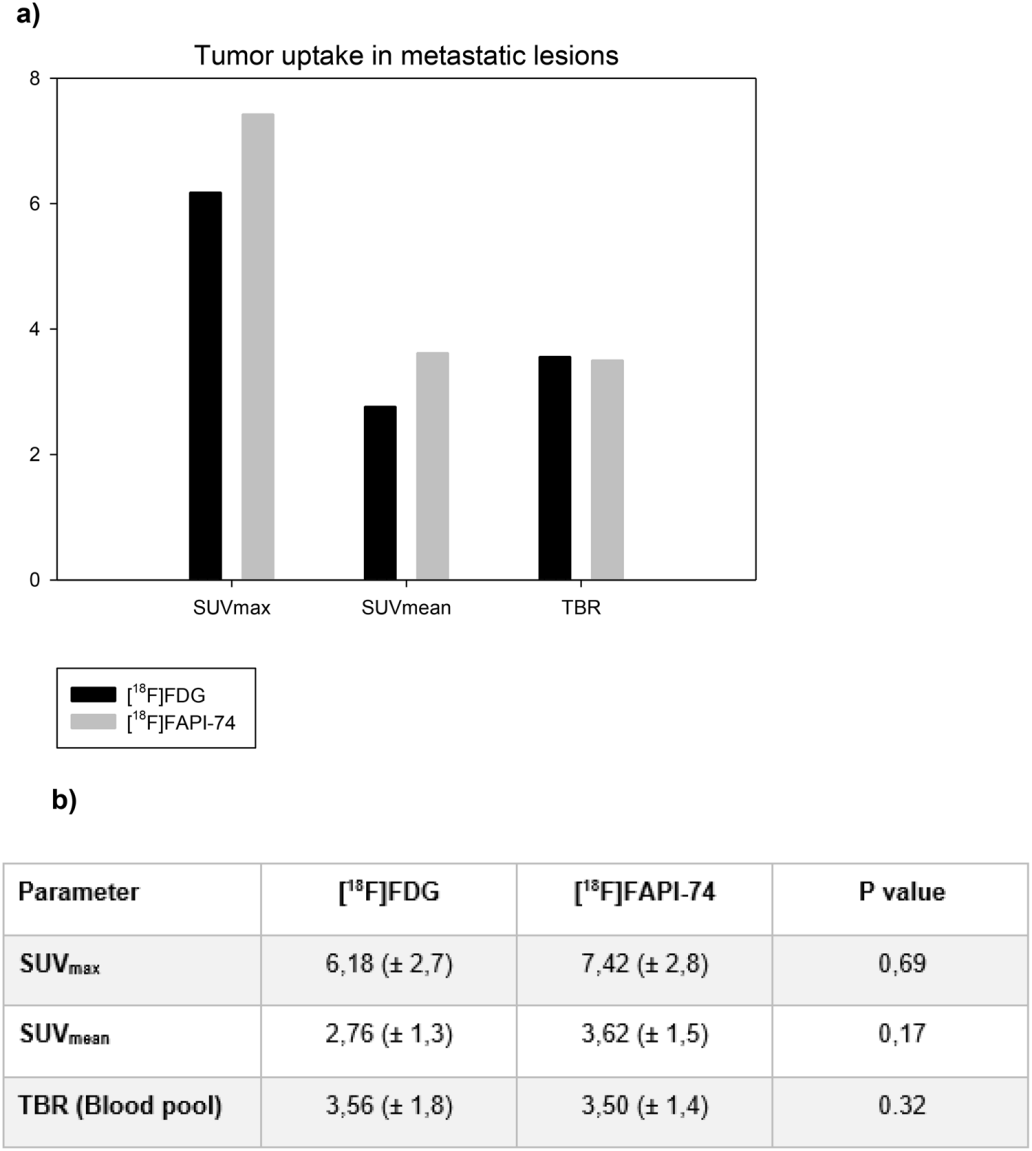
Tumor uptake of [^18^F]FAPI-74 and [^18^F]FDG for the metastatic lesions with means of SUV-metrics (**a**). Despite more favorable outcome for [^18^F]FAPI-74, there was no statistically significant difference among the results (**b**)

We observed a similar tendency also in the analysis of SUV-metrics (e.g. SUV_max_ ± SD, 7.42 ± 2.8 vs. 6.18 ± 2.7, [^18^F]FAPI-74 vs. [^18^F]FDG, respectively) in metastatic lesions. However, despite more favorable tracer uptake kinetics, SUV-metrics analysis did not reveal statistically significant results (Fig. 4a & b). Given the substantially higher number of detected metastatic lesions by [^18^F]FAPI-74, we presume that the results might be biased, as [^18^F]FAPI-74 outnumbered the detected metastatic lesions in [^18^F]FDG imaging by approximately 45%. In this regard, [^18^F]FDG imaging seemed to exhibit relatively favorable TBR and lesion delineation for the fewer detected lesions. Those lesions were mostly located in lymph nodes ([^18^F]FAPI-74 vs. [^18^F]FDG) (26 vs. 16) followed by lung (8 vs. 7), liver (5 vs. 3), bone (2 vs. 2) and peritoneum (1 vs. 1), noting that metastatic lesion classification relied on a composite reference standard (Table 2).

**Table 2:** Summary of the detected primary and metastatic lesions according to each imaging modality

Tumor Entity	[^18^F]FDG		[^18^F]FAPI-74	
	Primary Lesion (*n*)	Metastatic Lesion (*n*)	Primary Lesion (*n*)	Metastatic Lesion (*n*)
PDAC	6	17	7	21
STS	4	2	4	5
CRC	0	8	1	10
Gastric cancer	0	2	0	6
	10	29	12	42
Total (n)	39		54	

### The added value of [^18^F]FAPI-74 imaging

The cohort mostly consisted of patients with advanced tumor stage even during diagnostic work-up for primary staging. Consequently, the clinical impact of [^18^F]FAPI-74 imaging in terms of tumor stage change might be regarded as redundant for the majority of patients. Thus, we observed the real added value of [^18^F]FAPI-74 imaging with respect to per-lesion based detection rate, imaging contrast and accuracy of tumor delineation. These aspects play indeed a pivotal role in the therapy management of such patients, as they need target-lesion-oriented, patient-tailored palliative surgical or radio-therapeutical interventions.

**Fig. 5:**
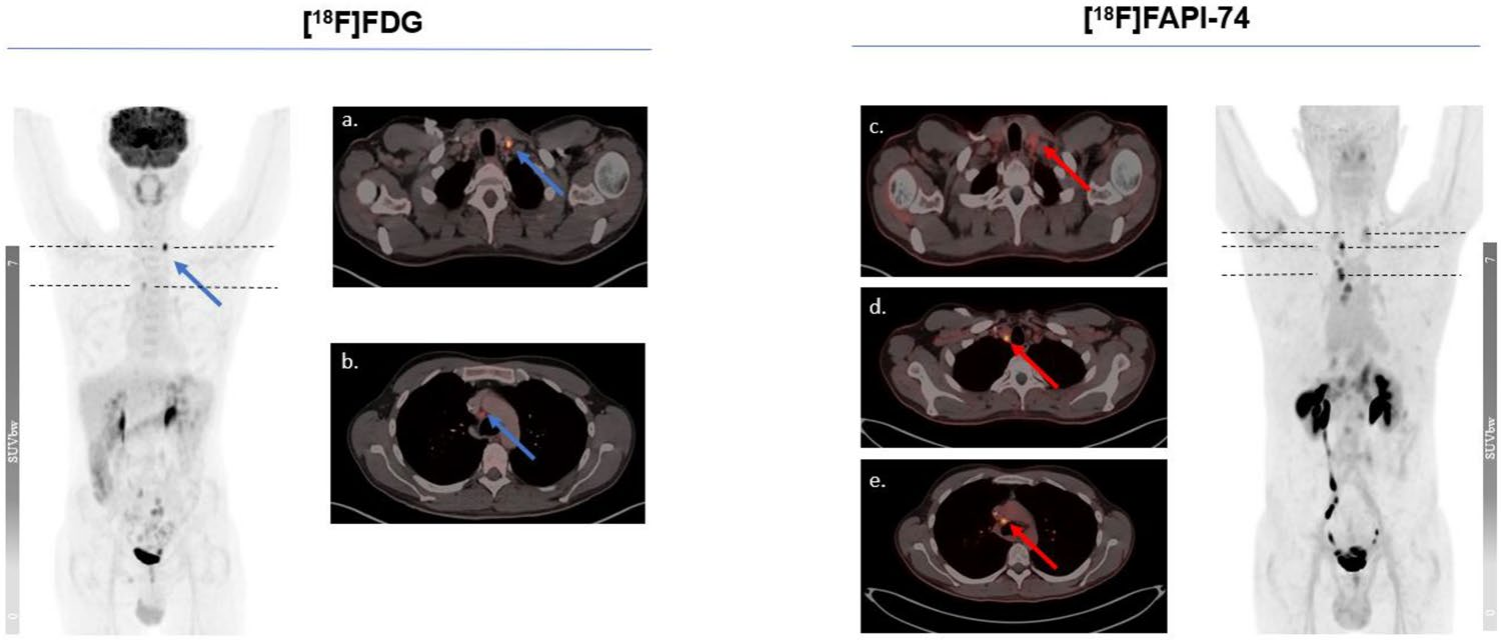
Intra-individual comparison in a 41-year-old male patient due to recurrence-work-up of gastric cancer. [^18^F]FAPI-74 enabled a superior diagnostic performance on per-lesion based assessment such as identification of lymph node metastases in neck or mediastinal region, albeit there was no change in tumor staging

**Fig. 6:**
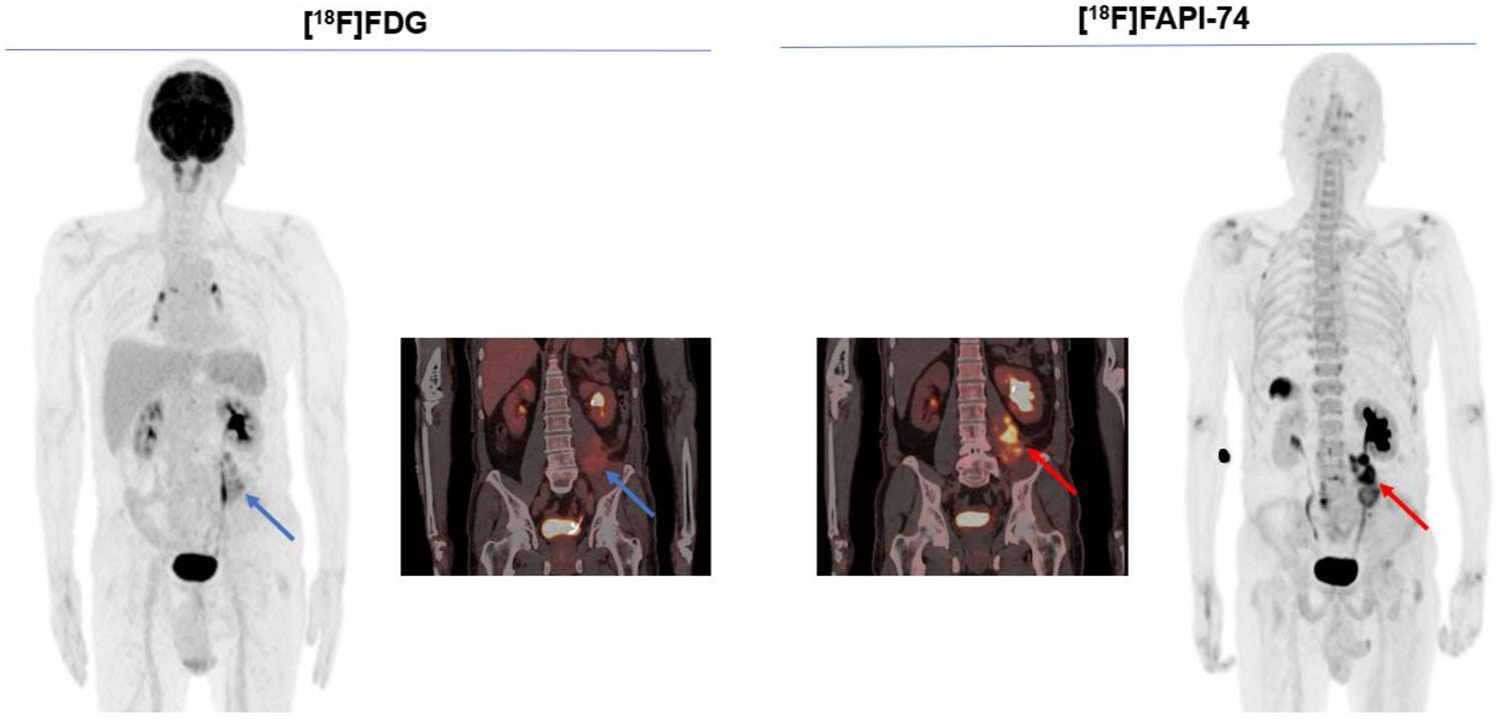
A 61-year-old male patient underwent dual-tracer imaging as a part of therapy response monitoring following radiotherapy due to myofibroblastic sarcoma of left distal psoas muscle. The further follow-up examinations validated a partial response, as this was also better delineated by [^18^F]FAPI-74 imaging than [^18^F]FDG imaging. Notably, gallbladder displayed [^18^F]FAPI-74 accumulation in accordance with biodistribution

**Fig. 7:**
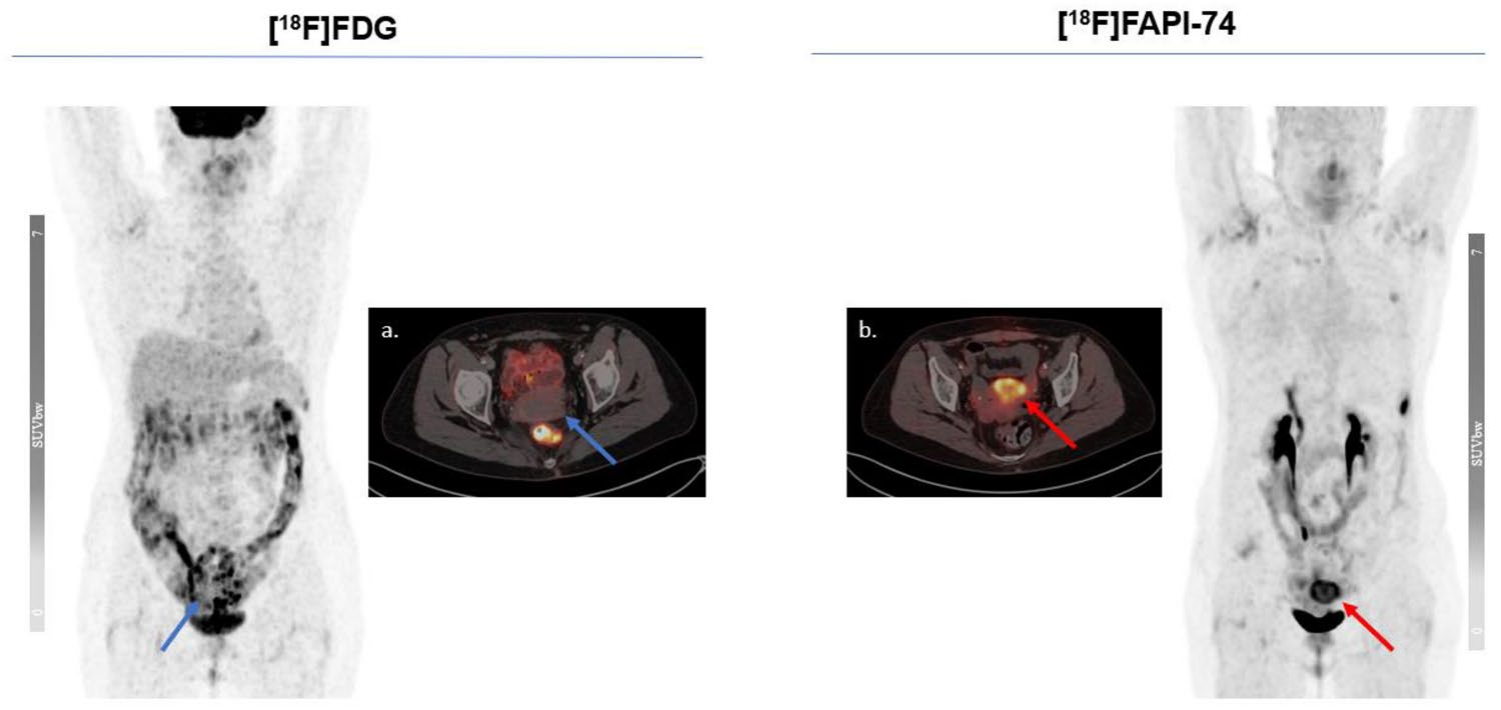
Locally advanced primary lesion of a rectum adenocarcinoma in a 60-year-old-female patient could only be revealed by [^18^F]FAPI-74. The [^18^F]FAPI-74 PET/CT scan demonstrated no further suspected lesions

The Figs. 5 and 6 represent typical examples outlining the added value of [^18^F]FAPI-74 imaging in oncological imaging of advanced stage malignancies. This provided a better lesion contrast owing to a better TBR and higher per-lesion-based detection rate. Figure 7 illustrates the case of a 60-year-old female patient with diagnostic work-up due to locally advanced, suspected rectal cancer. While the primary lesion showed no relevant [^18^F]FDG uptake, [^18^F]FAPI-74 delineated the primary lesion with a higher accuracy and favorable contrast.

## Discussion

This prospective, exploratory study with head-to-head comparison of [^18^F]FAPI-74 and [^18^F]FDG PET/CT imaging aimed to evaluate the imaging characteristics of [^18^F]FAPI-74 imaging in gastro-intestinal tumors including gastric, pancreatic (PDAC) and colorectal cancer (CRC) and soft tissue sarcomas (STS). The cohort encompassed 19 patients with histopathologically confirmed tumor entities in advanced stage, of which most underwent PET studies due to recurrence and re-staging work-up. This study was initiated shortly after the introduction of [^18^F]FAPI-74 by the Heidelberg research group. The primary focus was to investigate the imaging characteristics of this tracer in terms of biodistribution and tumor uptake pattern across different tumor entities that were already known to exhibit moderate to high FAP expression. The initial assessment of this tracer was conducted by the inventors in patients with lung cancer (Giesel et al. 2021).

The study design emphasized the feasibility of [^18^F]FAPI-74 for the abovementioned tumor entities in patients at an advanced TNM stage. The relatively small cohort size remains, however, as a key limitation of the study. Therefore, the study design did not allow the assessment of diagnostic performance in terms of sensitivity, specificity or predictive values. In addition, even though all tumor entities were biopsy-proven, the suspected metastatic lesions detected on PET studies could only be corroborated by composite reference method based on correlate on concordant follow-up imaging and clinical course.

The FAP imaging by ^68^Ga-labelled FAPI tracers have meanwhile provided convincing results for several epithelial malignancies. Particularly, the tumor entities with prominent tumor stroma or desmoplastic reaction appeared to be the most optimal entities for FAPI imaging, as these have been shown to express FAP not only in tumor stroma, but also on tumor cells (Fitzgerald and Weiner 2020, Gao et al. 2024, Kessler 2023). Currently, one of the main focuses of FAPI research is to generate evidence regarding the utility of ^18^F-labelled FAPI tracers in order to compensate the drawbacks of ^68^Ga-labelling such as limited cost-effectiveness and spatial resolution. However, the literature data regarding the utility of [^18^F]FAPI-74 PET is still very scarce.

Our data revealed a more favorable biodistribution of [^18^F]FAPI-74 compared to [^18^F]FDG in normal tissue and especially in metastasis-target organs. In addition, the tumor uptake of [^18^F]FAPI-74 in numerical SUV-metrics values including TBR was higher than that of [^18^F]FDG in the total malignant lesions, both in primary and metastatic lesions, across all investigated tumor entities. [^18^F]FAPI-74 imaging outperformed [^18^F]FDG imaging by 20% higher detection rate of primary lesions in addition to a better lesion delineation and imaging contrast as well. For the primary lesions, the increased TBR of [^18^F]FAPI-74 was statistically significant, whereas there was no difference of TBR and other SUV-metrics for metastatic lesions between the tracers. Given possible non-oncologic FAPI uptake in small or atypical lesions and the lack of histological validation, misclassification bias cannot be excluded in our cohort. The heterogeneity of suspected metastatic lesions and, in particular, increased number of detected lesions by FAPI imaging might explain the abovementioned discrepancy in tracer uptake pattern of primary and metastatic lesions. [^18^F]FAPI-74 imaging detected 45% more metastatic lesions than [^18^F]FDG (Table 2).

STS represents traditionally one of the most challenging family of malignancies due to a vast amount of molecular and clinical heterogeneity with varying levels of metabolic activity. The conventional methods including [^18^F]FDG imaging could not overcome these challenges, as FAP imaging with ^68^Ga-labelled FAPI tracers have been shown to enable a more personalized therapy management (Giammarile et al. 2024). Our results seemed to confirm these previous results, as our cohort also demonstrated a better diagnostic performance of [^18^F]FAPI-74 in terms of higher lesion detection rate and better tumor delineation. For example, the case of 61-year-old-male-patient with myofibroblastic sarcoma of left distal psoas muscle depicts the efficacy of [^18^F]FAPI-74 imaging very well. This indicated an unequivocal [^18^F]FAPI-74 uptake of the primary lesion following radiotherapy compared with [^18^F]FDG imaging. On the contrary, the faint [^18^F]FDG uptake in the psoas muscle left might be interpreted as post-radiotherapeutical changes in regular clinical care, which would have required at least a follow-up imaging for validation.

Gastrointestinal tumours including colorectal carcinoma, gastric carcinoma and pancreatic carcinoma are among the most lethal and prevalent malignancies worldwide with a limited diagnostic efficacy of [^18^F]FDG imaging. [^18^F]FDG imaging has namely low sensitivity in the detection of primary lesions and lymph node staging. Moreover, given the high physiological background of normal organs, [^18^F]FDG imaging has a limited diagnostic efficacy in detection of frequently encountered distant metastases like hepatic and peritoneal metastases (Zhuang et al. 2024, Pang et al. 2021, Hu et al. 2025). On the other hand, FAPI imaging has been demonstrated to provide highly promising results regarding the accurate detection and delineation of such lesions either due to favourable TBR due to lacking FAPI uptake in liver or owing to the fact that the growth of peritoneal metastasis follows a strong fibrotic response (Zhuang et al. 2024, Pang et al. 2021, Hu et al. 2025).

Consistent with the aforementioned literature data, our cohort had a 36% higher detection rate for malignant lesions than [^18^F]FDG imaging. Furthermore, [^18^F]FAPI-74 provided therapy relevant changes in diagnosis in 23% of patients. Our study outcome exhibited a high concordance with the report of Watabe et al., who conducted a prospective, single-center study with [^18^F]FAPI-74 in an intra-individual comparison to [^18^F]FDG imaging, underscoring the favorable biodistribution and tumor uptake leading to a good diagnostic outcome for GI tumors (Watabe et al. 2023).

## Conclusion

In this prospective, exploratory study cohort, [^18^F]FAPI-74 appeared to display more favorable imaging characteristics such as lesion contrast and delineation compared with [^18^F]FDG. Given the advantages of ^18^F-labeling, [^18^F]FAPI-74 warrants further investigation with larger cohorts to determine its diagnostic potential and clinical impact.

## Supplementary Information

Below is the link to the electronic supplementary material.

Supplementary Material 1

## Data Availability

The data used and/or analyzed during the current study are available from the corresponding author on reasonable request.

## Abbreviations

CAF: Cancer-associated-fibroblasts
CRC: Colorectal carcinoma
FAP: Fibroblast-Activation-Protein
FAPI: Fibroblast-activation-protein-inhibitor
[^18^F]AlF-FAPI-74: [^18^F]FAPI-74
MIP: Maximum intensity projection
PDAC: Pancreatic adenocarcinoma
STS: Soft tissue sarcoma
TBR: Tumor-to-background ratio
