# Supplementary material for: The analysis of biodistribution and tumor uptake of [18F]AlF-FAPI-74 in patients with soft tissue sarcoma and gastro-intestinal tumors compared with [18F]FDG in a prospective, exploratory study
Source: EJNMMI Rep. 2026 Mar 6;10(1):8. doi: 10.1186/s41824-026-00295-7 (PMC12963560; doi:10.1186/s41824-026-00295-7)
Supplement: Supplementary file 1 — Supplementary Material 1 [file 41824_2026_295_MOESM1_ESM.pdf]

Supplemental Table S1: Acquisition Protocol

**Site**	**Positronmed, Santiago, Chile**	
PET/CT scanner	Biograph Vision 450 (6R/64CT), Siemens	Biograph mCT Flow 20-3R
CT reference (mAs)	190	170
CT peak kilovoltage (kV)	100	140
CT slice thickness (mm)	2	2
CT slice increment (mm)	1	2
PET reconstruction	OSEM algorithm (PSF & TOF)	OSEM algorithm (PSF & TOF)
Iterations	4	2
Subsets	5	21
Matrix	220 x 220	200 x 200
Corrections	Gaussian FWHM 4.0 mm	Gaussian FWHM 4.0 mm
